# Antibiomania: a case report of clarithromycin and amoxicillin-clavulanic acid induced manic episodes separately

**DOI:** 10.1186/s12888-021-03397-7

**Published:** 2021-08-11

**Authors:** Edith Paula Meszaros, Catheline Stancu, Alessandra Costanza, Marie Besson, François Sarasin, Guido Bondolfi, Julia Ambrosetti

**Affiliations:** 1 Departments of Emergency and Psychiatry, Emergency Psychiatric Unit (UAUP), Geneva University Hospitals (HUG), Geneva, Switzerland; 2 Department of Psychiatry, Faculty of Medicine, University of Geneva (UNIGE), Rue Gabrielle-Perret-Gentil 4, 1205 Geneva, Switzerland; 3 Unit of Psychopharmacology, Department of Pharmacology and Toxicology, Geneva University Hospitals (HUG), Geneva, Switzerland; 4 Department of Emergency, Emergency Medicine Unit, Geneva University Hospitals (HUG), Geneva, Switzerland; 5 Department of Psychiatry, Service of Liaison Psychiatry and Crisis Intervention (SPLIC), Geneva University Hospitals (HUG), Geneva, Switzerland

**Keywords:** Antibiomania, Amoxicillin-clavulanic acid, Clarithromycin, Antibiotics, Mania, Case report, Adverse effects

## Abstract

**Background:**

Antibiomania is a rare but recognized side effect with yet unclear definite pathogenesis although multiple hypotheses have been proposed. The novelty of this case is the suspected pharmacodynamic drug-drug interaction between clarithromycin and amoxicillin-clavulanic acid.

**Case presentation:**

We present the occurrence of a brief manic episode concerning a 50-year-old man with no psychiatric history, first started on amoxicillin-clavulanic acid therapy and then switched to clarithromycin for left basal pneumonia. Shortly after the antibiotic prescription, he presented psychiatric symptomatology (logorrhea, elevated mood, irritability, increase in physical activity and delusions). The antibiotic was stopped and the patient received lorazepam (2.5 mg p.o.) to treat psychomotor agitation. Approximately 12 h after clarithromycin cessation, amelioration was already observed, supporting the diagnosis of a clarithromycin-induced manic episode. Amoxicillin-clavulanic acid was then reintroduced because of the pneumonia and psychiatric symptoms reemerged. This second antibiotic was also stopped, and 1 week later, the patient was symptom-free.

**Conclusion:**

The emergence of psychiatric side effects related to antibiotherapy, which is a common treatment, can greatly impact a patient’s quality of life. Early recognition and intervention could substantially influence the administered medical care and recovery. Moreover, given the widespread use of antibiotics including in combination, we thought our case report might be clinically useful as a clinical reminder relevant to the use of antibiotic combinations.

## Background

The term antibiomania first appeared in 2002, referring to manic symptomatology induced by antibiotics [1]. Despite numerous case reports, specific knowledge about this phenomenon is still lacking. Because its impact on the patient’s quality of life is relevant, it would be useful to know more about the individual susceptibility and arrive at a clinical consensus about managing these cases. The problem with reporting only this side effect to authorities in charge is that the clinical details are left out, hence the importance of published case reports [1].

Two systematic literature reviews [1, 2] synthetized most existent data on antibiomania by including 47 spontaneous case reports and 143 cases reported to the Food and Drug Administration (FDA) and World Health Organization (WHO). These authors reported that clarithromycin, ciprofloxacin, and ofloxacin were the most common causative antibiotics [1, 2]. The key diagnostic feature for antibiomania is considered to be the close temporal relationship between the administration of the antibiotic and the emergence of manic symptoms. The first and the most important intervention was the discontinuation of the causative agent, which leads to the remission of the symptoms without the addition of antimanic agents in 18 cases (39%) [2]. Concerning putative etiopathogenetic underlying mechanisms of antibiomania, several theories have been put forward. The main hypothesis assumed that most antibiotics such as clarithromycin, fluoroquinolones, and beta-lactam antibiotics, antagonize postsynaptic GABA-A receptors in neurons and may cause an increase in neuron excitability [3–5].

This case report is aimed to describe a patient presenting with a first manic episode while being on antibiotics for a pneumonia, in order to remind fellow practitioners about the possible psychiatric adverse effects of antibiotics having a major impact on a patient’s quality of life. The early recognition of this phenomenon is important for an optimized medical care.

## Case presentation

A 50-year-old male patient with no somatic or psychiatric history was diagnosed with left basal pneumonia at a hospital consultation and was started on amoxicillin-clavulanic acid (1 g t.i.d.), a beta-lactamase inhibitor antibiotic. Then, he was discharged. Due to persistent respiratory symptoms, the patient returned to the hospital the day after, and the treatment was switched to a macrolide antibiotic, clarithromycin (500 mg b.i.d.).

Two days after the second antibiotic prescription, his family members noted a progressive change in his behavior, with unusual logorrhea, irritability, increased physical activity, elevated mood, and ideas of being in contact with God. Due to these sudden changes, the family brought him back to the hospital. He was transferred to the emergency department (ED) of the University Hospital of Geneva (HUG), 2 days after modifying the antibiotic therapy. There were no known allergies, no tobacco, alcohol, illicit substance or daily medication intake (including steroids), and the patient had never taken antibiotics before.

At our first psychiatric evaluation, the patient reported that during the night after the introduction of the first antibiotic (amoxicillin-clavulanic acid), he had the feeling of dying and developed auditory hallucinations, as he was hearing God who was talking to him and saying he was chosen for a special mission. The psychiatric examination revealed a familiar attitude, a lightly increased psychomotor activity, irritability, logorrhea, elevated mood, difficulty falling asleep, as well as auditory hallucinations and mystic mood-congruent delusions that were, however, partially criticized. The antibiotic was immediately stopped and the patient received lorazepam (2.5 mg p.o.) as a treatment for psychomotor agitation. We chose to begin the psychopharmacotherapy very gradually, without immediate recourse to antipsychotic treatment, because the patient was partially nosognosic and the increased psychomotor agitation as well the irritability was foregrounded. Moreover, 12 h after clarithromycin cessation, amelioration was rapidly observed with the progressively gained insight of delusions and cessation of auditory hallucinations. The patient agreed that if his clinical conditions symptomatology did not improve, antipsychotic treatment would be necessary. These symptoms lasted in total about 36 h. The Young Mania Rating Scale (YMRS) score at the moment of the evaluation was 28, the equivalent of a manic episode [6].

The patient spent one night at the psychiatric ED observation unit of the HUG. After the above initial described amelioration during the first 12 h after the clarithromycin cessation, diminution of other manic symptoms was observed. Only logorrhea was still present. The YMRS score was 13, the equivalent of hypomanic symptomatology [6]. We discharged the patient with two tablets of lorazepam (1 mg p.o.). Considering the rapid amelioration of his symptoms by the antibiotics cessation and the only utilization of benzodiazepines, we chose not to initiate antipsychotics. We advised the patient to consult his family doctor in a few days and come back for a psychiatric evaluation 1 week later. Given the course of the clinical presentation the diagnosis of clarithromycin-induced manic episode was retained, and amoxicillin-clavulanic acid (625 mg b.i.d.) (Augmentin®) was then reintroduced for the pneumonia.

Investigations were completed with blood tests, which were in normal range except for high C-reactive protein at 200 mg/L without leukocytosis, rapid nasopharyngeal test for influenza A, B, and RSV, as well as cerebral scan, lumbar puncture and EEG. None of the results were contributive.

Since the patient was 50 years old, without psychiatric history, considering the close temporal relationship between the clarithromycin intake and the manic episode in addition to the rapid amelioration after clarithromycin cessation, the diagnostic of clarithromycin-induced manic episode was retained at this point as an exclusion diagnostic. We considered this event to be a probable adverse drug reaction based on the WHO-UMC system for standardized case causality assessment [7], and the Naranjo algorithm, which showed a score of 5, indicative of a probable causal reaction [8].

One week later, at the psychiatric follow-up evaluation, the patient presented the subjective impression of a mildly elevated mood and talkativeness and an YMRS score of 3, the equivalent of a euthymic state.

In fact, the patient reported a worsening of the psychiatric symptomatology the evening after the reintroduction of amoxicillin-clavulanic acid. As he left the hospital, he took two doses of 625 mg as prescribed. At midnight, he started hearing voices again, he felt persecuted and anxious. He was again partially nosognosic. The next day, he presented symptoms similar to the first manic episode, with logorrhea, difficulties sleeping, familiar attitude, and dysphoria. The patient took the two tablets of lorazepam (1 mg) he received (one during the night after leaving ED and one the next day). He called his family doctor, who recommended the cessation of the antibiotic and concluded an adverse reaction to amoxicillin-clavulanic acid. This evolution then oriented towards a pharmacodynamic drug-drug interaction between clarithromycin and amoxicillin-clavulanic acid, leading to antibiomania.

This case was reported to the national pharmacovigilance center, and the need for local ethics committee approval was waived.

## Discussion

Antibiomania can affect a wide age-range of the cases (from 3 to 77 years old, mean 40 years old), mostly arises in the absence of psychiatric history, and two-thirds of the patients are male [2]. The duration of the symptomatology lasted on average 3 days for the cases induced by clarithromycin in the published reports reviewed by Abouesh and colleagues [1]. Even though the exact incidence remains unknown, antibiomania is still a clinically rare phenomenon if we consider the significant augmentation in the worldwide consumption of antibiotics, which increased by 36% between 2000 and 2010 [9]. For this reason, although antiobiomania is reported in the literature to be a rare effect, many more cases should be described given the extremely common use of antibiotics.

The case of the patient presented here indicates an association between the prescription of amoxicillin-clavulanic acid and clarithromycin and the onset of manic symptoms, which is supported by the close chronology between the two antibiotics treatments and the development of a manic symptomatology, as well as by the data from the literature. In a systematic review of 47 cases, macrolides were found to be the incriminating agents in 18 cases, the same number of cases being induced by antituberculars, seven by quinolones, and two by beta-lactams [2]. In another review of spontaneous reports, also using data from World Health Organization (WHO), it was found that out of 82 cases, clarithromycin was implicated in 23 (27.6%), ciprofloxacin in 12 (14.4%), ofloxacin in ten (12%), isoniazid in five (6%), and amoxicillin-clavulanic acid in two (2.4%) cases [1]. The same review analyzed 21 reports of antimicrobial-induced mania found in the literature: six cases implicated clarithromycin, 13 implicated isoniazid, and one case each implicating erythromycin and amoxicillin.

In summary, macrolides, quinolones, and antitubercular drugs seem to be the most frequent group of antibiotics associated with mania. They all share a decrease in gamma aminobutyric acid (GABA) [2]. The systematic review realized by Lambrichts and colleagues [2], summarizes several etiopathogenetic hypotheses: quinolones competitively inhibit binding of GABA to their receptor sites; isoniazid, an antitubercular drug, inhibits the enzyme called glutamic acid decarboxylase (GAD); and clarithromycin, a macrolide antibiotic, antagonizes postsynaptic GABA-A receptors in neurons and may cause an increase of neuron excitability. Interestingly enough, even though the frequency of antibiomania is much lower for beta-lactams, they could also present a concentration-dependent GABA antagonism. In our case, the patient could have presented two separate manic episodes with two different antibiotics. However, because of the common mechanism of increasing neuron excitability and the almost concomitant assumption, a pharmacodynamic drug-drug interaction between amoxicillin-clavulanic acid and clarithromycin was also suspected. These hypotheses could explain the pharmacodynamic drug-drug interaction between amoxicillin-clavulanic acid and clarithromycin in the present case [5].

In addition to the action on GABA neurotransmission, there are other hypotheses to explain antibiomania. These hypotheses implicate an increased concentration of cortisol, prostaglandin E1 (PGE1), pro-inflammatory cytokines, or C-reactive protein, as well as the glutaminergic pathways, abnormalities in mitochondrial function induced by antibiotics and finally, associations between antibiotic administration and changes in the composition of microbiota in the gastrointestinal tract [2]. Further research is needed to clarify all the implicated mechanisms.

The respective role of each antibiotic during the episode is hard to elucidate, since the patient experienced auditory hallucinations after amoxicillin intake, which continued together with manic clinical signs with clarithromycin and the second course of amoxicillin. Nevertheless, a pharmacodynamic drug-drug interaction between the two antibiotics can be hypothesized since they are both known to trigger such an episode. A limitation of this case report is the lack of medical evaluation of the symptoms induced by the second course of amoxicillin because the patient did not return to the hospital.

Presumably, the patient presented here experienced psychiatric symptoms on two occasions, with two different antibiotics, highlighting the relevance of the individual biological susceptibility. There is a clear need for a close follow-up of the patients, especially at the first administration of antibiotics.

This case report needs to be interpreted in the light of two main limitations. First, there are already existing reports of cases of mania which develop after antibiotic use. Nevertheless, important knowledge is still lacking about this syndrome. Second, a rechallenge to verify our hypothesis of a pharmacodynamic drug-drug interaction between the two antibiotics was not done.

## Conclusion

Despite being a rare phenomenon, these clinical presentations and their differential diagnosis deserve greater attention, both for doctors working in primary care and mental health professionals. The emergence of psychiatric side effects related to antibiotherapy, which is a very commonly utilized treatment, can greatly impact on a patient’s quality of life. Early recognition and intervention could substantially influence the administered medical care and recovery. Moreover, given the widespread use of antibiotics, including in combination, we believe that our case report can be a clinically useful reminder relevant to antibiotic-combination treatments.

## Data Availability

Data sharing is not applicable to this article as no datasets were generated or analysed.

## Abbreviations

b.i.d.: bis in die (twice a day)
ED: Emergency Department
EEG: Electroencephalography
FDA: Food and Drug Administration
GABA: Gamma Aminobutyric Acid
HUG: University Hospitals of Geneva
PGE1: Prostaglandin E1
p.o.: per os
RSV: Respiratory-Syncytial-Virus
t.i.d.: ter in die (three times a day)
WHO: World Health Organization
YMRS: Young Mania Rating Scale
